# Ambulatory children with spastic cerebral palsy have smaller bone area and deficits in trabecular microarchitecture

**DOI:** 10.1093/jbmr/zjaf026

**Published:** 2025-02-10

**Authors:** Elizabeth A Zimmermann, Louis-Nicolas Veilleux, Marianne Gagnon, Dominique Audet, Rita Yap, Catherine Julien, Seyedmahdi Hosseinitabatabaei, Eliane Rioux Trottier, Bettina M Willie, Alessandra Carriero, Jean-Pierre Farmer

**Affiliations:** Faculty of Dental Medicine and Oral Health Sciences, McGill University, Montreal (QC) H3A 0C7, Canada; Research Center, Shriners Hospitals for Children, Montreal (QC) H4A 0A9, Canada; Department of Surgery, McGill University, Montreal (QC) H4A 3J1, Canada; Research Center, Shriners Hospitals for Children, Montreal (QC) H4A 0A9, Canada; Department of Surgery, McGill University, Montreal (QC) H4A 3J1, Canada; Department of Clinical Research, Shriners Hospitals for Children, Montreal (QC) H4A 0A9, Canada; Department of Physiotherapy, Shriners Hospitals for Children, Montreal (QC) H4A 0A9, Canada; Research Center, Shriners Hospitals for Children, Montreal (QC) H4A 0A9, Canada; Faculty of Dental Medicine and Oral Health Sciences, McGill University, Montreal (QC) H3A 0C7, Canada; Research Center, Shriners Hospitals for Children, Montreal (QC) H4A 0A9, Canada; Department of Biomedical Engineering, McGill University, Montreal (QC) H3A 2B4, Canada; Research Center, Shriners Hospitals for Children, Montreal (QC) H4A 0A9, Canada; Department of Surgery, Centre Hospitalier Universitaire Sainte-Justine, Montreal (QC) H3T 1C5, Canada; Faculty of Dental Medicine and Oral Health Sciences, McGill University, Montreal (QC) H3A 0C7, Canada; Research Center, Shriners Hospitals for Children, Montreal (QC) H4A 0A9, Canada; Department of Biomedical Engineering, McGill University, Montreal (QC) H3A 2B4, Canada; Department of Pediatric Surgery, McGill University, Montreal (QC) H4A 3H9, Canada; Department of Biomedical Engineering, The City College of New York, New York (NY) 10031, United States; Research Center, Shriners Hospitals for Children, Montreal (QC) H4A 0A9, Canada; Department of Pediatric Surgery, McGill University, Montreal (QC) H4A 3H9, Canada

**Keywords:** cerebral palsy, bone density, gait analysis, HRpQCT, bone microarchitecture

## Abstract

Cerebral palsy (CP) is a non-progressive neurological syndrome resulting in abnormal muscle tone, movement, and posture. It is unclear whether ambulatory children with CP have deficits in bone quantity or quality. Furthermore, the relationship between abnormal muscle tone, altered function, and bone health remains largely unexplored. This observational study investigated bone mineral density (BMD) and microarchitecture in ambulatory children with spastic CP and associations of BMD with function, muscle spasticity, and gait. Children with spasticity in both lower limbs (*n* = 12) aged 3-8 years were recruited. Areal BMD was measured with dual energy x-ray absorptiometry (DXA) at the proximal femur and lateral distal femur and compared to normative data. High resolution peripheral quantitative computed tomography (HR-pQCT) was performed at the metaphyseal tibia and radius in a subset of participants (*n* = 5) and compared to healthy children (*n* = 7). Gait pathology and cardiopulmonary function were investigated with the Gait Deviation Index, Edinburgh Visual Gait Score, and energy expenditure index. DXA areal BMD (aBMD) *Z*-scores at the lateral distal femur were within a normal range. However, the CP group’s median aBMD *Z*-score at the proximal femur was −1.8 (IQR: −2.2, −1.2, *p* = .03) indicating potential skeletal fragility. Strong correlations were found between gait pathology and DXA-based bone outcomes (correlation coefficient 0.62 [*p* = .04] to 0.73 [*p* = .01]) as well as energy expenditure index and DXA-based bone outcomes (correlation coefficient −0.63 [*p* = .03] to −0.98 [*p* ≤ .001]). At the metaphyseal tibia, children with spastic CP had significant deficits in HR-pQCT-measured bone geometry and trabecular microarchitecture: 35% lower total area, 42% lower trabecular area, and 48% lower trabecular number than controls. HR-pQCT parameters were similar between groups at the metaphyseal radius. These differences in tibial metaphysis size and trabecular microarchitecture are similar to those observed in disuse and thus could be a result of abnormal biomechanics or low levels of physical activity.

## Introduction

Cerebral palsy (CP) is a non-progressive childhood neurological disorder caused by injury of the immature brain. CP is the most common motor disability in children with an estimated prevalence around 2.5 per 1000 live births.^1^ Individuals with CP exhibit abnormal muscle tone (eg, spasticity, hypotonia), movement, and posture that results in a progressive musculoskeletal disorder (ie, bone deformity, joint contractures, and hip subluxation). However, the relationship between abnormal muscle contractions, altered mobility, and bone health remains largely unexplored at present.

CP is commonly related to prematurity, low birth weight, hypotension, hypoxia, or ischemia, leading to periventricular leukomalacia.^2^ The type and severity of impairment varies widely and depends on the part of the brain that is injured. Spastic CP, the most common form occurring in 82% of cases, is characterized by velocity dependent increases in muscle tone (ie, stiffness or shortening of muscles).^3^ Spasticity in children with CP can lead to abnormal gait patterns (eg, toe walking, jump knee, crouch knee, and stiff knee)^4^ and reduced mobility (ie, use of a walker or wheelchair to move in the community and/or at home). Children with CP are classified in terms of limitations in motor function with the Gross Motor Function Classification System (GMFCS).^5^ In GMFCS level I, children walk with no assistance, level II walk without assistance but have limitations in balance and long distance walking, level III use a mobility device (eg, walker) to walk long distances in most settings, level IV use a mobility device to walk short distance but use a wheelchair for transport over long distances; and level V rely on a mobility device for all settings due to limited voluntary control of movement.

Recent studies have shown that adults with CP have musculoskeletal morbidity. Adults with CP (18-64 years) have a nearly 3-fold greater prevalence of bone fractures than individuals without CP, especially in the lower extremities.^6^ Greater prevalence of fractures starts in adolescence and continues through adulthood^7^; fractures are distributed across all GMFCS levels.^8^ Furthermore, age groups as young as 18-30 years have a 7 times greater odds of musculoskeletal morbidity (osteoporosis, osteopenia, osteoarthritis) than individuals without CP.^9^

Longitudinal bone growth as well as bone modeling and remodeling are influenced by mechanical forces.^10^^,^^11^ Thus, spasticity, abnormal gait, and limitations in mobility could affect bone growth and metabolism. Although children with CP have normal skeletal characteristics at birth,^12^^,^^13^ children at all GMFCS levels can develop foot and lower limb bone deformities.^14–16^ Abnormal gait patterns caused by spasticity are related to alterations in lower limb geometry.^15^ Our previous skeletal growth studies have demonstrated how altered gaits in children with CP apply abnormal loads on the developing bones,^17^ thus leading to long bone deformities.^18^

Bone mass has been investigated in children with CP using dual energy x-ray absorptiometry (DXA), which is a convenient clinical tool. While the International Society for Clinical Densitometry (ISCD) defines osteoporosis in the pediatric populations as a DXA Z-score $\le$ −2.0 and a clinically significant history of fracture,^19^^,^^20^ the ISCD position is that a Z-score > −2.0 in children may still be associated with skeletal fragility.^19^^,^^20^ Using DXA, there is no evidence to-date of low aBMD at the proximal femur in children ambulating independently in the community (ie, GMFCS levels I and II); however, there is evidence for low bone density in children using a wheelchair for most activities or only ambulating in the household (GMFCS level IV, V).^21^^,^^22^ At the lateral distal femur, Finbraten et al.^23^ observed low bone density (Z-score < −2) in GMFCS level II but not GMFCS level I; however, Chen et al.^24^^,^^25^ observed significantly lower aBMD at the lateral distal femur in ambulatory children with CP but the Z-score was greater than −1.

Here, in ambulatory children aged 3-8 years with spastic CP predominantly in the lower limbs, we investigate bone density and bone microarchitecture with DXA and high resolution peripheral quantitative computed tomography (HR-pQCT). Compared to DXA, HR-pQCT has a number of advantages; most importantly, it separates the cortical and trabecular bone and measures bone microarchitectural parameters in each compartment. Furthermore, we investigate associations between bone outcomes and measures of spasticity, function, and gait.

## Materials and methods

### Ethical considerations

This study was approved by the McGill University Institutional Review Board under authorizations #A04-M25-20B (participants with CP and three controls) and A06-M28-19B (HR-pQCT data for four additional controls who were scanned in the same manner as the CP participants). Informed consent was obtained from parents and a pictorial assent was used to explain the study to participants, as they were 8 years and younger.

### Study design

Children with CP were recruited for a prospective observational study to investigate the effects of selective dorsal rhizotomy surgery on bone and gait outcomes over 12 months. The target sample size was based on power considerations for change in aBMD Z-score at the lateral distal femur. Here, we report the baseline (pre-operative) bone and gait outcomes in the CP cohort. HR-pQCT was also performed on a control group of children without CP because normative data are unavailable for this age range.

### Participants

Participants were recruited from the spasticity clinic at the Shriners Hospital for Children—Canada and from children of staff for controls. The inclusion criteria for children with CP were the following: (1) age of 3-8 years, (2) clinical diagnosis of spastic CP, (3) GMFCS I, II, III, or IV, (4) candidate for selective dorsal rhizotomy surgery, (5) planned rhizotomy surgery in the next 12 months, and (6) no botulinum toxin injections in the 6 months prior to baseline. Besides the other inclusion criteria, children with CP were candidates for selective dorsal rhizotomy surgery if they had sufficient strength underlying spasticity, good trunk control, and spasticity limiting progress in gross motor skills and ambulation.

The inclusion criteria for the non-CP control group were the following: (1) age of 3-8 years, (2) no clinical presentation of CP or other movement disorders, (3) no history of use of an assistive device for ambulation, (4) no history of bed rest, and (5) no bone fracture or orthopedic surgery in the 12 months prior to baseline.

The exclusion criteria for both groups were the following: (1) history of familial spastic paraplegia or paraparesis, (2) confounding skeletal or other medical conditions that might interfere with the objectives of the study, (3) indications interfering with imaging (eg, rods in limbs, limb fracture in last 2 years near imaging site), (4) use of prescription medication targeting growth, bone mineral accrual or spasticity, (5) current or prior use of a baclofen pump.

### Demography and medical history

The following data were collected from the electronic medical file and through interview with the parents: sex, age, race, ethnicity, GMFCS level, and use of assistive devices for ambulation, presentation of spasticity and/or other movement disorders, fracture history, botulinum toxin injection history, orthopedic interventions, and pharmaceutical therapies. Weight measured in kilograms (kg) and standing height in centimeters (cm) was measured prior to imaging by a nurse. Weight-for-age and height-for-age Z-scores were calculated with the child growth standards (for children 0 to 5 years) and the growth reference data for 5 to 19 years from the World Health Organization.^26^

### Dual-energy x-ray absorptiometry

aBMD (g/cm^2^) was measured with a DXA scanner (Hologic QDR Discovery) at the proximal femur and lateral distal femur of the non-dominant leg. The proximal femur scan occurred with the child in a supine position. The lateral distal femur scan occurred with the child lying on their side with the leg of interest lying straight against the scanner bed and the other leg bent in a comfortable position. The lateral distal femur is used in the CP population because supine positioning is not always possible due to muscle contractures and the femur is also a common fracture site.^27^^,^^28^

**Figure 1:**
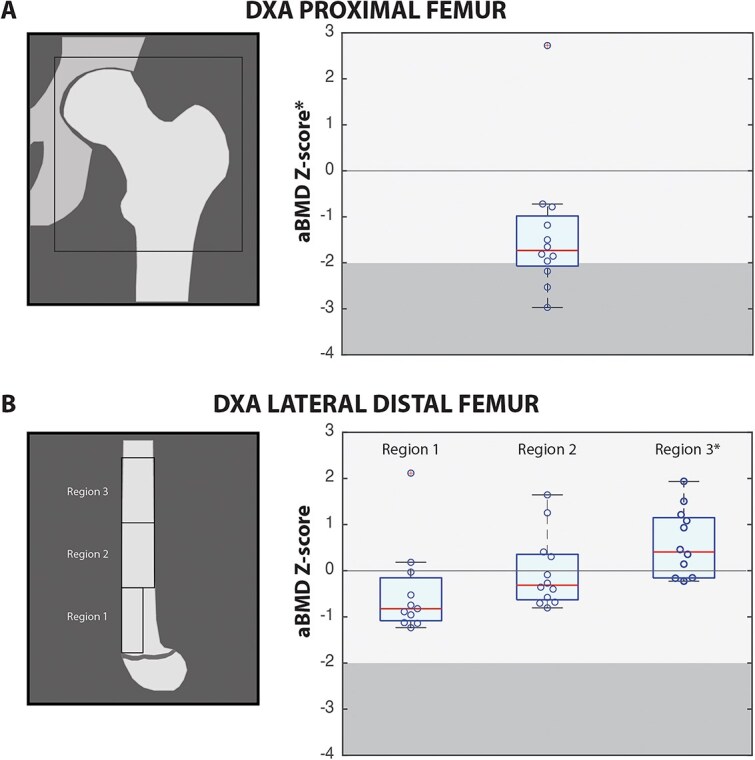
Areal bone mineral density (aBMD) in ambulatory children with spastic CP. Dual-energy x-ray absorptiometry (DXA) was used to measure aBMD and aBMD *Z*-scores at the non-dominant (A) proximal femur and (B) three regions in the non-dominant lateral distal femur. Boxplots for aBMD *Z*-scores show the median value (line within box), upper and lower quartile values (top and bottom of box), maximum and minimum data values (whiskers), and outliers (circle inset with a cross outside of box and whiskers). The median aBMD *Z*-score at the proximal femur and the mean lateral distal femur *Z*-score for region 3 were significantly different from zero (indicated by *). The darker shaded region indicates a *Z*-score $\le$−2.0, which together with a clinically significant history of fracture, represents osteoporosis in the pediatric population according to the ISCD.^19^^,^^20^ However, the ICSD’s position is that a *Z*-score > −2.0 in children may still be associated with skeletal fragility.^19^^,^^20^

The lateral distal femur analysis separated the scanned area into three regions by using the following procedure. First, the diameter of the femur near the diaphysis was measured. Then, three regions were constructed each with a length in the proximal-distal direction of twice the diaphyseal diameter. Region 1 abutted both the growth plate and the anterior femur with a width of one half the distal femur width. Regions 2 and 3 were located proximal to Region 1 and had widths equal to the full diameter of the bone (Figure 1). Region 1 represents predominantly trabecular bone, Region 3 represents predominantly cortical bone, and Region 2 will be a mix of cortical and trabecular bone.^28^ The outcome parameters were aBMD at the proximal femur and each region of the lateral distal femur. Z-scores were calculated using normative data published in the literature for children between the ages of 3-18 years.^27^^,^^29^ For the lateral distal femur, there are two pediatric normative datasets that overlap between the ages of 3-12 years; we chose to use the dataset of Henderson et al. (2002) because it covers a wider age range.^27^^,^^29^

### High resolution peripheral quantitative computed tomography

Bone density and microarchitecture were investigated using a XtremeCT II scanner (Scanco Medical). The length of the tibia was measured by the radiological technician as the distance between the medial malleolus and the tibial plateau. The length of the ulna was measured by the radiological technician between the proximal end of the olecranon process and the distal end of the styloid process. Following the manufacturer’s imaging protocols, for radius measurements, the child’s non-dominant arm was placed in the manufacturer’s pediatric arm restraint. For tibia measurements, the adult leg restraint was used with padding to secure the non-dominant leg. Throughout the test, the lights were dimmed, and the child was instructed to remain still like a statue during the approximately 6-min scan.

The volume of interest (VOI) for each scan was placed using a scout view of the distal portion of the radius or tibia. All participants in this study had visible growth plates and the reference lines were placed at the most distal margin of the growth plate. The metaphyseal tibia stack was acquired starting at 4% of the tibial length.^30^ The metaphyseal radius scan was acquired starting at 4% of the ulnar length.^30^ The VOIs consisted of a double stack (ie, two stacks of 168 slices each) covering approximately 20.4 mm in length at 60.7-μm isotropic voxel size. Scans with a motion artifact score greater than 3 were excluded.^31^

To process and analyze the HR-pQCT data, the manufacturer’s standard reconstruction script was implemented. Then, the auto-contouring function was used to find automatically the outer contour of the bone; the placement of the outer contour was checked and corrected manually if necessary. Next, the standard data analysis script from the manufacturer was run followed by verification of the endocortical contours and manual correction, if necessary. The standard analysis calculated total area (Tt.Ar) and total volumetric BMD (Tt.vBMD) as well as the following cortical bone parameters: Cortical volumetric BMD (Ct.vBMD), area (Ct.Ar), thickness (Ct.Th), and porosity (Ct.Po). Trabecular parameters included trabecular volumetric BMD (Tb.vBMD), area (Tb.Ar), bone volume fraction (Tb.BV/TV), thickness (Tb.Th), number (Tb.N), and separation (Tb.Sp).

### Gross Motor Function Measure-66 and modified Ashworth scale

The Gross Motor Function Measure (GMFM) is the gold standard for measuring change in motor function in children with CP comprising of five dimensions^32^: (1) lying and rolling, (2) sitting, (3) crawling and kneeling, (4) standing, and (5) walking, running and jumping. The GMFM-88 provides dimension scores and a total score. The GMAE-2 software (distributed by CanChild) was used to convert the GMFM-88 to GMFM-66 total score. The conversion from GMFM-88 to GMFM-66 was done with a formula of proven reliability.^32^

The modified Ashworth scale was also performed by a physiotherapist to describe muscle tone in lower limb muscle groups (hip adductors, knee flexors, knee extensors, and ankle plantar flexors). The scale was from 0 to 4, where 0 indicates no spasticity and 4 indicates rigidity (no passive movement).

### Edinburgh visual gait score

The Edinburgh Visual Gait Score (EVGS) provided a validated method to quantitatively assess gait quality on video recording in CP.^33^ The EVGS included 17 items that assessed the position of the foot, knee, hip, and trunk at different phases of the gait cycle. Each item was scored 0, 1, or 2 based on the deviation from normal population for which 0 is normal.

### Energy expenditure index

The energy expenditure index (EEI) was measured during a 6-minute walk test.^34^ Participants (*n* = 9) walked at a self-selected speed around a 14.5-meter oval for 6 minutes using their regular walking device and wearing a heart-rate belt monitor (Polar Electro Oy). The heart rate and the walking distance were used to calculate the EEI using the following formula, and therefore to assess the walking efficiency.


$$ \mathrm{EEI}=\frac{\mathrm{walking}\ \mathrm{heart}\ \mathrm{rate}-\mathrm{resting}\ \mathrm{heart}\ \mathrm{rate}}{\mathrm{walking}\ \mathrm{velocity}} $$

### Quantitative gait analysis

Quantitative gait analysis was performed on collaborative participants (*n* = 10) to measure kinematics (dynamic joint ranges of motion).^35^ The kinetics were not collected on most participants due to walking aids (walker, canes) or steps that were too small. Kinematics were captured at 100 Hz using a 10-camera motion-capture system (Vicon Vantage V8; Oxford Metrics) and using the Plug-in-Gait model. Participants walked at a self-selected speed along a 10-meter walkway. Data were collected and processed with Nexus software version 2.12 for foot strike and toe-off identification (Oxford Metrics). Data were imported in Matlab (version 2021a) for analysis. The gait parameters were used to calculate the gait deviation index, which is a measure of gait pathology in children with CP.^36^ A child with no gait abnormalities would have a score of 100 $\pm$10.

### Statistics

All statistical analyses were performed in SPSS 29.0.0.0 (IBM SPSS Statistics). For function, gait, and DXA outcomes, the data were first tested for normality using the Shapiro–Wilk’s test. Data are presented as mean ± SD with 95% confidence intervals, median, and interquartile range. One outlier for DXA at the lateral distal femur, Region 1 was excluded because the aBMD values were unrealistically low and not reproducible at the follow-up appointment (follow-up data not reported here). DXA *Z*-score at Region 3, gait deviation index, EVGS, and energy expenditure index were normally distributed; thus, an one-sample *t*-test was performed to test the following null hypotheses: the mean *Z*-score is equal to zero, the mean gait deviation index is equal to 100, the mean EVGS is equal to 0, and the mean energy expenditure index is equal to 0.48.^34^ DXA *Z*-scores at the proximal femur and lateral distal femur Regions 1 and 2 were not normally distributed; thus, a one-sample Wilcoxon Signed Rank Test was performed to test the null hypothesis that the median *Z*-scores are equal to zero.

Correlation of DXA bone outcomes with function and gait measures were assessed with Pearson correlation for continuous, normal data and with Spearman’s rho for continuous, non-normal data. Kendall’s tau was performed to explore associations between continuous bone parameters and ordinal GMFCS level as well as continuous bone parameters and ordinal Modified Ashworth Scale.

For the HR-pQCT data, the data were first tested for normality and homoscedasticity using the Shapiro–Wilk’s test. Levene’s test was performed to test for equal variances. For data passing normality, an independent *t*-test was performed between control and CP groups. For data not passing normality, the Mann–Whitney test was performed. Analysis of covariance (ANCOVA) was performed to compare HR-pQCT parameters between CP and control groups after controlling for the height of participants. Scatterplots were visually inspected to determine if there was a linear relationship between HR-pQCT parameters and height. Shapiro–Wilk’s test was used to test normality of the standardized residuals for the individual CP and control groups as well as for the overall model. Homogeneity of variance was assessed with Levene’s test. Outliers, homoscedasticity, and homogeneity of regression slopes were checked. Most HR-pQCT parameters met all the assumptions of the ANCOVA; however, if assumptions were not met, the adjusted *p*-value is not reported.

## Results

### Characteristics of the cohort

The characteristics of the CP cohort are presented in Table 1. Children with CP exhibited spasticity in the lower limbs (Table S1). An exclusion criterion for participating in the study was use of medication targeting bone mineral accrual; nonetheless, some medications used by the participants may affect bone metabolism. One participant used the anti-convulsant clobazam, levetiracetam, and gabapentin, which negatively impact bone.^37^ Other medications used included the following (*n* = 1 participant unless indicated otherwise) with a beneficial (+), negative (−), or unknown (?) effect on bone health: laxatives (?) (*n* = 2), acetaminophen (−), NSAIDs (−), melatonin (?) (*n* = 2), vitamin D (+), proton pump inhibitors (−), probiotics (?), and inhalers (−) (*n* = 2).

**Table 1:** Characteristics of the CP cohort.

**CP cohort characteristics**	
	***n* = 12**
**Sex**	
**Male**	11
**Female**	1
**Age at enrollment (years)**	
**Mean (SD)**	5.3 (0.8)
**Range**	3.8-6.3
**Standing height (cm)**	
**Mean (SD)**	102.0 (5.5)
**Standing height *Z*-score**	
**Mean (SD)**	−2.0 (0.6)
**Weight (kg)**	
**Mean (SD)**	16.6 (2.5)
**Weight *Z*-score**	
**Mean (SD)**	−1.1 (0.9)
**GMFCS**	
**I**	0
**II**	4
**III**	7
**IV**	1
**Spasticity distribution**	
**Diplegia**	10
**Triplegia**	2
**Assistive device for ambulation** ^**a**^	
**(yes:no)**	10:2
**Previous botulinum toxin injection** ^**a**^	
**(yes:no)**	6:6
**Previous orthopedic interventions** ^**a**^	
**(yes:no)**	1:11
**Previous medications** ^**a**^	
**Antispastic/muscle relaxers (yes:no)**	1:11
**Anticonvulsants (yes:no)**	1:11
**Anticholinergics (yes:no)**	0:12
**Antiresorptives (yes:no)**	0:12
**Other (yes:no)**	6:12
**Previous bone fracture** ^**a**^	
**(yes:no)**	0:12

a Use or event in the past 2 years.

Abbreviations: CP, cerebral palsy; GMFCS, Gross Motor Function Classification System; SD, standard deviation.

### DXA-measured deficits in bone mass at proximal femur but not lateral distal femur

Table 2 reports the aBMD and aBMD *Z*-scores at the proximal femur and lateral distal femur for the CP cohort (*n* = 12). The median aBMD *Z*-score at the proximal femur was significantly lower than zero. Median lateral distal femur *Z*-scores for Region 1 and 2 were not significantly different from zero (Table 2, Figure 1). However, the mean lateral distal femur *Z*-score for Region 3 was significantly higher than zero, with a portion of *Z*-scores more than one standard deviation greater than the norm.

**Table 2:** Descriptive statistics for aBMD, function, and gait in the CP cohort.

	**Mean ± SD**	**Median**	**Interquartile range**	**95% CI**	***p*-Value**
**Proximal femur** ^**a**^ **: aBMD (gHA/cm** ^**2**^ **)**	0.506 ± 0.114	0.481	[0.438, 0.518]	[0.433, 0.579]	NA
**Proximal femur** ^**a**^ **: aBMD *Z*-score**	−1.4 ± 1.4	−1.8	[−2.2, −1.2]	[−2.3, −0.4]	**.03**
**Later distal femur: aBMD (gHA/cm** ^**2**^**)**					
**Region 1**^**a**^	0.637 ± 0.104	0.602	[0.579, 0.668]	[0.567, 0.707]	NA
**Region 2**^**a**^	0.682 ± 0.080	0.662	[0.627, 0.706]	[0.631, 0.733]	NA
**Region 3**	0.752 ± 0.064	0.742	[0.696, 0.836]	[0.711, 0.793]	NA
**Lateral distal femur: *aBMD Z-score***					
**Region 1**^**a**^	−0.5 ± 1.0	−0.8	[−1.1, 0.0]	[−1.1, 0.2]	.08
**Region 2**^**a**^	0.0 ±0.8	−0.4	[−0.7, 0.4]	[−0.5, 0.5]	.58
**Region 3**	0.6 ± 0.7	0.4	[−0.2, 1.2]	[0.1, 1.0]	**.02**
**Function and gait**					
**GMFM-66 score**	54.7 ± 8.4	55.7	[53.6, 65.6]	[49.3, 60.0]	NA
**Gait deviation index**	56.9 ± 10.2	53.0	[48.5, 67.0]	[49.6, 64.2]	**<.001**
**Edinburgh visual gait score**	40.3 ± 9.6	39.5	[26.8, 45.5]	[34.2, 46.5]	**<.001**
**Energy expenditure index (beats/m)**	1.78 ± 1.03	1.26	[1.03, 1.93]	[0.99, 2.57]	**<.01**

Data are given as mean $\pm$SD, median, interquartile range, and the 95% confidence interval (CI). *p*-Values reported for one-sample *t*-test or one-sample Wilcoxon Signed Rank Test for parametric and non-parametric data, respectively. The null hypothesis tested is whether the mean (or median for non-parametric test) *Z*-scores are equal to zero, that the mean gait deviation index is equal to 100, that the mean Edinburgh Visual Gait Score is equal to 0, and that the mean energy expenditure index is equal to 0.48. Bold p-values are significant (*p* < 0.05). Abbreviations: aBMD, areal bone mineral density; CP, cerebral palsy; gHA, grams of hydroxyapatite; GMFM-66, gross motor function measure; NA, not applicable.

a Data are not normally distributed.

### Impairments in motor function and gait in the CP group

The GMFM-66 scores (*n* = 12) ranged from 42.1 to 70.8, which was within the range expected for children of this age group with GMFCS levels II-IV.^38^ The mean gait deviation index (*n* = 10) was significantly lower than 100 and indicated gait pathology in the CP cohort (Table 2); it is expected that children with CP would have a gait deviation index that deviates from normal values.^36^ Similarly, the mean EVGS (*n* = 12) was significantly greater than zero, which also indicated gait pathology in the CP cohort (Table 2). The mean energy expenditure index (*n* = 9) (Table 2) was significantly greater than the published normative value of 0.48 beats/min for children 6-8 years; thus, the average cardiovascular energy used during gait in the CP cohort was higher than the norm.^34^ Quantitative gait analysis (to measure gait deviation index) and energy expenditure index were not measured in the full cohort because the children were either fatigued or uncooperative with the process.

### Gait deviation index and energy expenditure index correlate with bone outcomes

Motor function and spasticity in the lower limbs could impact bone mass. Thus, associations were investigated between measures of bone mass and motor function. GMFCS level classified children with CP according to their limitations in mobility; however, there was no significant correlation between aBMD and GMFCS level. GMFM-66 described motor function abilities and can be used to track how motor function changes over time. There was no significant correlation between aBMD and GMFM-66 score (Table 3). Abnormal muscle tone was quantified through the Modified Ashworth Scale. Weak associations between spasticity scores measured by Modified Ashworth Scale in lower limb muscle groups were found (Table S2). However, a negative significant correlation was observed between the Modified Ashworth Scale in the left knee extensors and aBMD at the proximal femur. Similarly, a negative significant correlation was observed between the Modified Ashworth Scale in the right knee extensors and aBMD *Z*-scores at the lateral distal femur Region 1 (Table S2). Thus, higher muscle spasticity in the knee extensors was associated with lower bone density at the proximal femur and Region 1 of the lateral distal femur.

**Table 3:** Correlation coefficients of bone density with gait and function.

	**GMFCS level**	**GMFM-66 Score**	**Edinburgh visual gait score**	**Gait deviation index**	**Energy expenditure index (EEI)**
**aBMD proximal femur**	−0.18 (0.48)	0.32 (0.15)	0.03 (0.46)	0.27 (0.23)	−**0.98 (<0.001)**
***Z*-score proximal femur**	−0.10 (0.69)	0.27 (0.20)	0.03 (0.47)	0.20 (0.29)	−**0.90 (<0.001)**
**aBMD lateral distal femur**					
**Region 1**	0.37 (0.16)	−0.31 (0.18)	0.32 (0.17)	**0.62 (0.04)**	−0.26 (0.27)
**Region 2**	−0.28 (0.27)	0.42 (0.09)	−0.10 (0.38)	0.25 (0.24)	−**0.64 (0.03)**
**Region 3**	−0.28 (0.27)	0.39 (0.11)	−0.20 (0.26)	**0.67 (0.02)**	−**0.63 (0.03)**
**aBMD *Z*-score lateral distal femur**					
**Region 1**	0.42 (0.11)	−0.36 (0.14)	0.27 (0.22)	**0.73 (0.01)**	−0.21 (0.31)
**Region 2**	−0.18 (0.48)	0.21 (0.26)	−0.04 (0.45)	0.22 (0.27)	−0.40 (0.14)
**Region 3**	−0.14 (0.58)	0.09 (0.40)	−0.12 (0.36)	**0.62 (0.03)**	−0.44 (0.12)

Data are shown as the correlation coefficient (*p*-value). Pearson correlation performed on continuous, parametric data (aBMD and *Z*-score lateral distal femur Region 3). Spearman’s rho performed on continuous, non-parametric data. Kendall’s tau performed to explore associations between continuous bone parameters and ordinal GMFCS level. Bold values are significant (*p* < 0.05). Abbreviations: aBMD, areal BMD; GMFCS, Gross Motor Function Classification System.

Gait deviation index describing the lower limb biomechanics was also associated with measures of bone mass (see Table 3). There was a strong positive significant correlation between gait deviation index to the aBMD and aBMD *Z*-scores at the lateral distal femur (Regions 1 and 3); this implies that less gait abnormality corresponds to higher bone density. Similarly, there was a very strong negative significant correlation between energy expenditure index and the aBMD *Z*-score at the proximal femur. There was also a strong significant negative correlation between the energy expenditure index and the aBMD at the lateral distal femur (Region 2 and 3). Thus, children with CP having a more efficient walking pattern exhibited greater bone density. There was no significant correlation between DXA data and EVGS.

### Smaller HR-pQCT-measured bone geometry and trabecular microarchitecture at metaphyseal tibia

A subgroup of children in the CP cohort underwent HR-pQCT at the metaphyseal radius and metaphyseal tibia. The characteristics of the subgroups are given in Table 4. Body weight and age were not significantly different between the groups, but the CP group was on average shorter than the control group. The full cohort was not measured for the following reasons: radiology not available, child’s limb was too short and could not fully enter the scanner, or the child was not cooperative or coughing.

**Table 4:** HR-pQCT data at metaphyseal radius and metaphyseal tibia.

			**Control group**			**CP group**			***p*-value**	***p*-value adjusted for height**
**Subgroup characteristics**										
**Sex**	Male:Female		4:3			4:1			–	
**Age**	Mean ± SD	years	5.9	±	1.2	4.7	±	0.7	.06	NA
	Range	years	4.8-7.7			3.8-5.4			–	
**GMFCS level**	I:II:III:IV		NA			0:1:3:1			–	
**Standing height**		cm	112.8	±	8.2	99.6	±	5.7	**.01**	NA
**Standing height *Z*-score**		–	–0.4	±	0.9	−1.9	±	0.8	**.01**	NA
**Weight**		kg	19.0	±	2.8	15.6	±	2.6	.06	NA
**Weight *Z*-score**		–	–0.5	±	0.6	−1.1	±	1.0	.22	NA
**Metaphyseal radius**			*n* = 7			*n* = 4				
**Ulnar length**		cm	16.9	±	1.3	15.6	±	1.1	.14	NA
**Total area**	Tt.Ar	mm^2^	93.2	±	12.5	79.7	±	11.1	.11	0.59
**Total vBMD**	Tt.vBMD	mg HA/cm^3^	358.3	±	37.3	327.2	±	64.9	.23	0.12
**Cortical vBMD**	Ct.vBMD	mg HA/cm^3^	796.8	±	29.2	730.4	±	54.8	**.03**	NR
**Cortical area**	Ct.Ar	mm^2^	30.1	±	4.5	27.3	±	0.8	.26	NR
**Cortical thickness**	Ct.Th	mm	1.034	±	0.112	0.966	±	0.100	.34	0.21
**Cortical porosity**	Ct.Po	mm^3^/mm^3^	0.004	±	0.002	0.004	±	0.001	1.00	NR
**Trabecular vBMD**	Tb.vBMD	mg HA/cm^3^	138.9	±	28.9	127.3	±	27.5	.53	0.99
**Trabecular area**	Tb.Ar	mm^2^	59.3	±	10.1	58.4	±	17.9	.92	0.08
**Trabecular bone volume fraction**	Tb.BV/TV	mm^3^/mm^3^	0.172	±	0.044	0.149	±	0.035	.40	0.88
**Trabecular number**	Tb.N	1/mm	1.351	±	0.298	1.122	±	0.276	.24	0.93
**Trabecular thickness**	Tb.Th	mm	0.193	±	0.011	0.199	±	0.010	.41	0.16
**Trabecular separation**	Tb.Sp	mm	0.739	±	0.190	0.912	±	0.208	.19	0.77
**Metaphyseal tibia**			*n*	=	7	*n*	=	5		
**Tibial length**		cm	23.6	±	2.7	19.6	±	1.3	**.01**	NA
**Total area**	Tt.Ar	mm^2^	290.7	±	46.8	186.6	±	31.5	**<.01**	**0.04**
**Total vBMD**	Tt.vBMD	mg HA/cm^3^	270.2	±	41.6	263.3	±	34.6	.76	0.86
**Cortical vBMD**	Ct.vBMD	mg HA/cm^3^	801.6	±	39.7	762.2	±	24.3	.20	0.34
**Cortical area**	Ct.Ar	mm^2^	58.2	±	11.4	45.1	±	4.0	**.04**	NR
**Cortical thickness**	Ct.Th	mm	1.110	±	0.173	1.138	±	0.103	.75	0.48
**Cortical porosity**	Ct.Po	mm^3^/mm^3^	0.006	±	0.004	0.006	±	0.003	.92	NR
**Trabecular vBMD**	Tb.vBMD	mg HA/cm^3^	126.1	±	25.6	75.9	±	23.9	**<.01**	0.07
**Trabecular area**	Tb.Ar	mm^2^	209.8	±	30.4	121.4	±	26.5	**<.001**	**0.02**
**Trabecular bone volume fraction**	Tb.BV/TV	mm^3^/mm^3^	0.172	±	0.036	0.111	±	0.029	**<.01**	0.14
**Trabecular number**	Tb.N	1/mm	1.260	±	0.210	0.657	±	0.188	**<.001**	**0.01**
**Trabecular thickness**	Tb.Th	mm	0.222	±	0.012	0.211	±	0.013	.18	0.54
**Trabecular separation**	Tb.Sp	mm	0.793	±	0.162	1.650	±	0.436	**<.01**	NR

HR-pQCT are collected as a double stack (336 slices). Data for individual stacks are presented in the supplement. Data are presented as unadjusted mean $\pm$SD, unless otherwise marked. An independent *t*-test or a Mann–Whitney test was performed. ANCOVA was performed to adjust the HR-pQCT parameters for height (if ANCOVA assumptions were violated, the adjusted *p*-values are not reported). Bold p-values are significant (*p* < 0.05). Abbreviations: Ct.Ar, area; Ct.vBMD, cortical volumetric BMD; Ct.Th, thickness; Ct.Po, porosity; GMFCS, Gross Motor Classification System; NA, not applicable; NR, not reported; SD, standard deviation.

The HR-pQCT data at the metaphyseal radius and metaphyseal tibia are presented for the full stack (336 slices) in Table 4 and separately for the proximal and distal stacks (168 slices each) in Table S3 and Table S4. Unadjusted data and *p*-values as well as *p*-values adjusted for height are presented. At the metaphyseal radius, there were no significant differences between the control and CP groups in bone geometry, cortical microarchitecture or trabecular microarchitecture. However, Ct.vBMD at the metaphyseal radius tended to be lower in the CP group compared to the controls (unadjusted *p*-values). Data for the full stack and the distal stack did not meet the assumptions of the ANCOVA (specifically, homogeneity of regression slope) and therefore, could not be adjusted for height. However, in the proximal stack, data were adjusted for height and significance of Ct.vBMD was lost. Future studies on larger cohorts are needed to confirm the results at the radius. It is important to note that 3/4 of the CP cohort were diplegic (spasticity only in lower limbs) and thus their arms were not affected by muscle spasticity.

**Figure 2:**
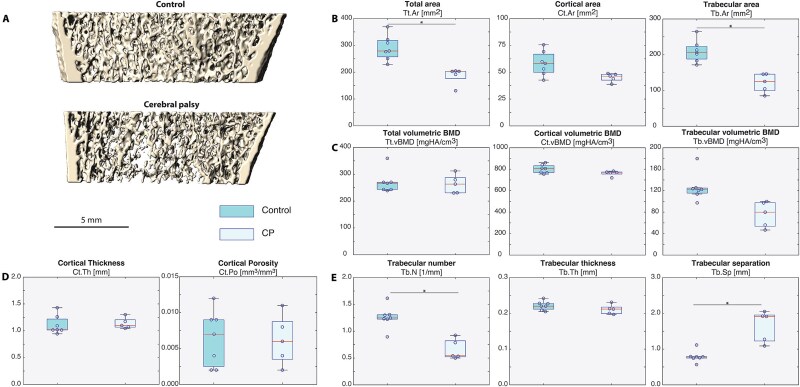
HR-pQCT data for metaphyseal tibia. Volumetric BMD and bone microarchitecture data are presented for the metaphyseal tibia in ambulatory children with spastic CP in both lower limbs in comparison to children without CP. (A) As shown in 3D representations of the HR-pQCT data from individuals from the control and CP groups, this data represent a double stack (supplement presents data separately for proximal and distal metaphyseal stacks). (B) Total area and trabecular area are significantly lower in the CP group. (C) Total, cortical, and trabecular vBMD are similar between the groups. (D) Cortical microarchitectural parameters were not signifciantly different between the groups. (E) Similarly, the CP group exhibited fewer trabeculae that are further apart (unadjusted *p*-value) with no difference in trabecular thickness. Boxplots show the median value (line within box), upper and lower quartile values (top and bottom of box), maximum and minimum data values (whiskers), and outliers (circle inset with a cross outside of box and whiskers). Significance was assessed with ANCOVA (to adjust for height) unless indicated. *p-value < .05.

Greater differences were observed between the groups at the metaphyseal tibia. As is apparent from the 3D reconstructions (Figure 2A) and 2D HR-pQCT slices (Figure S1), the bones are visibly smaller in diameter in CP participants. These differences in size were confirmed by area measures: the CP group had a 36% lower mean total area and a 42% lower mean trabecular area, which were both significant after adjusting for height (Figure 2B). There were no differences in total, cortical, or trabecular vBMD after adjusting for height (Figure 2C) as well as cortical microarchitecture (Figure 2D). Similarly, for the trabecular microarchitecture, the mean trabecular number is 47% lower in the CP group, which is accompanied by a 108% larger mean trabecular separation (unadjusted *p*-value) with no difference in trabecular thickness (Figure 2D). One tibia dataset from a participant with CP only contains one stack because the second stack contained motion.

Similar trends are observed at the tibia when the HR-pQCT data are analyzed as a double stack (Table 4) to when the HR-pQCT data are analyzed as individual proximal and distal stacks (Table S4). At the metaphyseal tibia, the total area is significantly higher in the metaphyseal distal stack of the control group, while the trabecular area is significantly greater in the proximal stack of the control group. Similarly, the same deficits in trabecular microarchitecture (Tb.N, Tb.Th) are observed when analyzing the stacks together or separately.

## Discussion

Here, we investigate bone density and bone microarchitecture as well as associations between bone outcomes and measures of spasticity, function, and gait in ambulatory children with spastic CP primarily affecting the lower limbs between the ages of 3 and 8 years. DXA measures of aBMD indicate trends toward low bone mass at the proximal femur but not at the lateral distal femur in children with spastic CP in both lower limbs. In the CP group, greater aBMD was associated with less gait pathology, lower energy expenditure, and less spasticity in the knee extensors. Even so, HR-pQCT imaging at the metaphyseal tibia showed smaller bones with deficits in trabecular microarchitecture compared to controls.

DXA is a practical tool used to measure bone mass in children. The International Society for Clinical Densitometry (ISCD) defines osteoporosis in the pediatric populations as a DXA *Z*-score $\le$−2.0 and a clinically significant history of fracture.^19^^,^^20^  *Z*-scores compare DXA aBMD values to normative values for a sex and age-matched population but it is not considered to be a reliable measure to predict fracture in children.^39^ Indeed, the ICSD’s position is that a *Z*-score > −2.0 in children may still be associated with skeletal fragility.^19^^,^^20^

Here, we observed a median aBMD *Z*-score of *Z* = −1.8 at the proximal femur, with some children below the *Z* = −2.0 threshold (Figure 1). Our data combining GMFCS levels II-IV are comparable to previous studies that found low bone density in children using a wheelchair for most activities or only ambulating in the household (GMFCS level IV, V) but not for children ambulating independently in the community (ie, GMFCS levels I and II).^21^^,^^22^ Although children in the CP cohort did not have any history of fracture in the preceding 2 years (Table 1), the ICSD’s position states that the children with *Z*-score > −2.0 may still be at risk of fracture.

Interestingly, the aBMD *Z*-scores at the lateral distal femur were normal to high, ie, more than one standard deviation greater than age and sex-matched normative values (Figure 1). In CP, various factors could contribute to abnormal bone mass and some aspects could either be protective or deleterious, such as spastic muscles, abnormal biomechanics, tendon properties, use of assistive devices for mobility, and level of physical activity. At the lateral distal femur, Finbraten et al.^23^ observed low bone density (*Z* < −2) in GMFCS level II but not GMFCS level I; however, Chen et al.^24^^,^^25^ observed significantly lower aBMD at the lateral distal femur in ambulatory children with CP but the *Z*-score was greater than −1.

Here, we found that greater DXA-based aBMD was associated with less gait pathology, less energy used while walking, and less spasticity in the knee extensors. There are few studies relating bone health and gait pathology for pediatric cohorts. More research is needed in this area to understand how bone density is affected by gait pathology or low levels of activity. We have previously used gait kinematics with quantitative motion analysis to differentiate walking patterns in CP^4^ and found a relationship between gait kinematics and lower limb morphology.^15^ We have previously demonstrated that abnormal gaits alter the loading conditions on bone,^17^ thus generating deformities.^18^

We find that HR-pQCT imaging provides a greater understanding of the bone phenotype in CP at a 61-μm voxel size over 10-20 mm of the bone’s length. Trabecular and cortical bone are segmented to measure volumetric BMD separately for each compartment as well as microarchitectural features (eg, cortical thickness, trabecular thickness). In contrast, DXA has an inferior spatial resolution to HR-pQCT, is size dependent, and is not capable of differentiating cortical and trabecular bone compartments.^40^

With HR-pQCT, children with CP were found to have smaller total and trabecular areas at the metaphyseal tibia than controls. Similar trends in cortical bone structure (for the outcomes of cortical area, total volume, cortical volume, medullary volume) were observed in the tibia and femur with pQCT and MRI in ambulatory and non-ambulatory children with CP.^41–43^ Our results are important because even in ambulatory children with a range of gait pathology and GMFCS levels II, III, and IV, the CP group has smaller bones and deficits in trabecular bone. Smaller bones are biomechanically disadvantageous. In skeletal regions subjected to bending, stresses are inversely proportional to the diameter to the fourth power (moment of inertia). In skeletal regions under compression, stresses are inversely proportional to the area of bone. Therefore, a smaller diameter bone will have a lower resistance to bending and compression than a larger diameter bone, which increases fracture risk.

The periosteal circumference of the tibia is visibly smaller in the children with CP compared to controls. Height-adjusted HR-pQCT outcomes found that the trabecular area and total area are significantly lower in the CP group compared to controls. The periosteal circumference of the tibia is determined by periosteal bone apposition.^44^ The differences in bone size and trabecular microarchitecture at the metaphyseal tibia are similar to those observed in disuse. Thus, the causes of this defective periosteal bone apposition should be investigated in CP. Possible causes include abnormal loading, low levels of physical activity, or a decreased response to loading, since periosteal bone formation occurs in response to loading.^45^

Exercise interventions show that bone mass in children with CP does respond to exercise.^46^ Weight bearing activities over 8 months in ambulatory and non-ambulatory children with CP significantly improved DXA-measured vBMD at the femoral neck.^47^ An at home cycling program improved lateral distal femur aBMD in ambulatory CP children after 12 weeks.^48^ Vibration plate training for 6 months in children with CP (GMFCS I-IV) improved tibial cortical bone area and moment of inertia measured with computed tomography.^49^ Thus, weight-bearing activities in children with CP may improve aBMD and cortical area.^46–49^ Further studies are warranted to investigate the relationship between bone density and aspects of mechanical loading (eg, physical activity level, gait pathology, reductions in spasticity). However, simply promoting physical activity in this population may be advantageous to their bone density. More importantly, physical activity should be investigated as a potent therapy to increase periosteal bone apposition and subsequent bone cross sectional area, to reduce fracture risk in these children.^50^

Our HR-pQCT results in the metaphyseal tibia further indicate that the trabecular vBMD and bone volume fraction are significantly lower in the CP group compared to controls; however, after adjusting for height, this comparison was only significant for trabecular vBMD in the proximal stack of the metaphyseal tibia (Table S3). The reason for this potential deficit in trabecular vBMD and bone volume fraction is a reduced trabecular number and subsequently an increased trabecular separation, while trabecular thickness was not significantly different. Further studies using HR-pQCT to measure cortical and trabecular BMD and microstructure on larger sample sizes are warranted. Thus, pharmacological therapies should be explored that will initiate the formation of new trabeculae to improve bone microstructural deficits.

This study is the first conducted in children with CP to analyze their bone properties using HR-pQCT, in comparison to DXA; however, the study has the following limitations. As a proof-of-concept study, it includes a small cohort of children. Recruitment for this study was hindered due to the global pandemic. Notably, the HR-pQCT data distinctly show a difference in bone size and trabecular microarchitecture in the CP group compared to healthy. This demonstrates potential changes in bone microarchitecture due to CP that are evident with HR-pQCT in comparison to DXA, which has clearly demonstrated limitations when using BMD *Z*-scores for prediction of fractures in children.^39^ This information is important and will inform future studies in this population. Future studies should include a larger cohort of CP children with HR-pQCT data to effectively determine their bone health.

Determining the appropriate control group is very difficult in children with CP due to their heterogeneous presentation (eg, type of CP, gait patterns, use of assistive devices, muscle tone patterns). This cohort was selected from children with CP eligible for rhizotomy surgery. Thus, the criteria for rhizotomy surgery (spastic diplegia, capacity for forward motion during ambulation, below 8 years of age) tend to reduce heterogeneity in this cohort. As cohorts with CP are heterogeneous, comparison to a healthy group or more severe cases (ie, non-ambulatory CP) show the general effect of the disorder, while comparison to themselves over time in a longitudinal study can further support the conclusions. Prospective studies should be considered in the design of future studies.

A potential limitation is socio-economic status between the control and CP groups, as the groups were recruited from different populations. The cohort is mostly male. The CP cohort was recruited from the population of children attending the spasticity clinic at Shriners and scheduled for an upcoming rhizotomy surgery; this population happened to be mostly male during our recruitment period. While there is a greater prevalence of CP in males than females,^1^ it will be important to also study females in the future.

The HR-pQCT data were acquired as a double-stack (single stack 168 slices, double stack 336 slices). Double stacks are often acquired in growing children to capture a greater length of bone to ensure matching at later timepoints in longitudinal studies. The limb lengths in the CP group were similar in the forearm but significantly smaller in the tibia. Thus, the volumes imaged with HR-pQCT are slightly different and exacerbated by the double stack. The length of the double stack is about 2 cm; thus, on average, roughly 8.5% of the metaphyseal tibia was imaged in the control group and 10.2% in the CP group. Additionally, factors such as age, sex, gender, race, and Tanner stage could affect bone outcomes.^40^

In conclusion, HR-pQCT measurements of bone size, vBMD, and microarchitecture indicate that children with spastic CP in both lower limbs have smaller bones with deficits in trabecular bone. HR-pQCT will be an important tool to further understand the bone phenotype in CP and its relationship to movement. Here, a strong correlation was found between gait pathology (gait deviation index) and DXA-based bone outcomes as well as energy expenditure index and DXA-based bone outcomes suggesting that improving gait patterns and cardiopulmonary fitness in ambulatory children with CP may improve their bone health. Further studies on larger sample sizes are justified to investigate bone mass phenotype in ambulatory children with CP and its relationship to movement and muscle activity.

## Supplementary Material

Baseline_bone_data_in_CP_17Mar2025_supplement_zjaf026

## Data Availability

The data underlying this article will be shared on reasonable request to the corresponding author.
