# Supplementary material for: Ambulatory children with spastic cerebral palsy have smaller bone area and deficits in trabecular microarchitecture
Source: J Bone Miner Res. 2025 Feb 10;40(4):511–21. doi: 10.1093/jbmr/zjaf026 (PMC12010165; doi:10.1093/jbmr/zjaf026)
Supplement: Baseline_bone_data_in_CP_17Mar2025_supplement_zjaf026 [file baseline_bone_data_in_cp_17mar2025_supplement_zjaf026.docx]

Supplementary figures and tables for:

**Ambulatory children with spastic cerebral palsy have smaller bone area and deficits in trabecular microarchitecture**

Elizabeth A. Zimmermann^1, †,*^, Louis-Nicolas Veilleux^2,3, †^, Marianne Gagnon^2,3^, Dominique Audet^4^, Rita Yap^5^, Catherine Julien^2^, Seyedmahdi Hosseinitabatabaei^1,2,6^, Eliane Rioux Trottier^2,7^, Bettina M. Willie^1,2,6,8^, Alessandra Carriero^9^, Jean-Pierre Farmer^2,8^

^1^Faculty of Dental Medicine and Oral Health Sciences, McGill University, Montreal, Canada

^2^Research Center, Shriners Hospitals for Children, Montreal, Canada

^3^Department of Surgery, McGill University, Montreal, Canada

^4^Department of Clinical Research, Shriners Hospitals for Children, Montreal, Canada

^5^Department of Physiotherapy, Shriners Hospitals for Children, Montreal, Canada

^6^Department of Biomedical Engineering, McGill University, Montreal, Canada

^7^Department of Surgery, Centre Hospitalier Universitaire Sainte-Justine, Montreal, Canada

^8^Department of Pediatric Surgery, McGill University, Montreal, Canada

^9^Department of Biomedical Engineering, The City College of New York, New York, USA

^†^These authors contributed equally

**Figure S1**


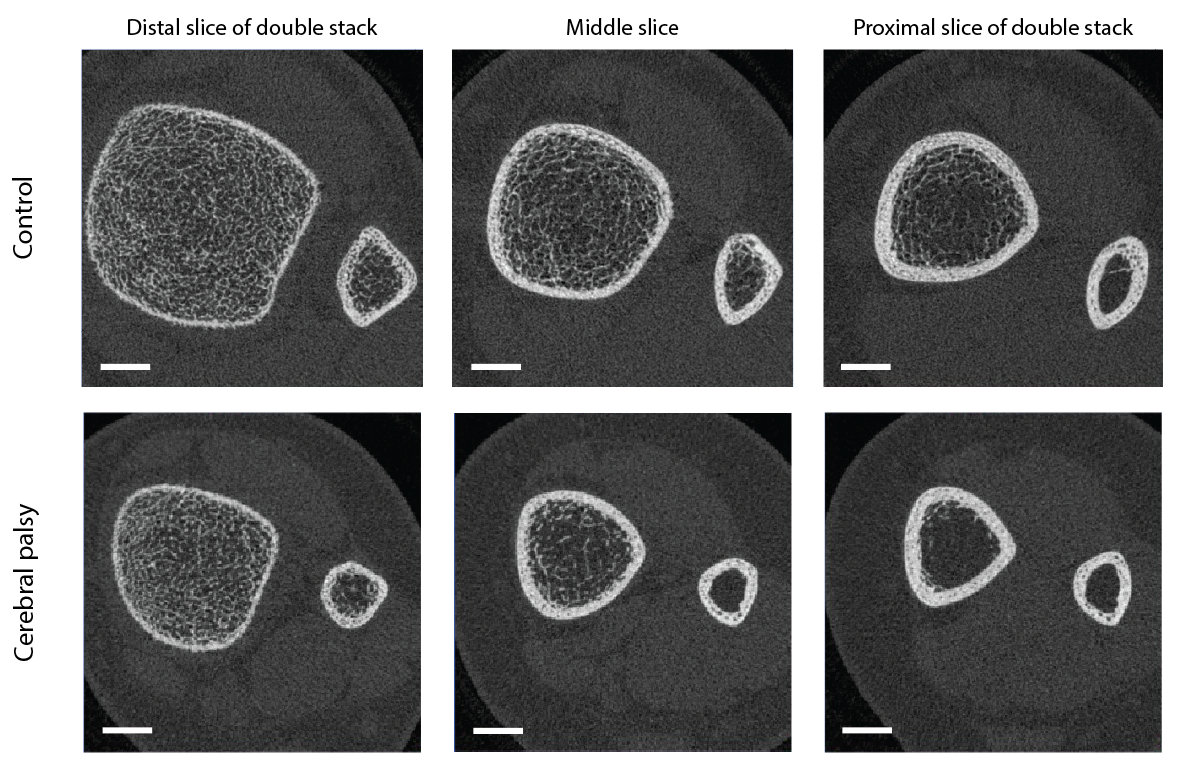


**Figure S1. *2D slices from HR-pQCT image stack of metaphyseal tibia.*** Here, 2D slices are shown from the HR-pQCT image stack from a participant from the control and cerebral palsy groups at the metaphyseal tibia. The HR-pQCT protocol consisted of a double stack (336 slices, 62 µm isotropic voxel size). The 2D images shown here are from the most distal slice of the double stack (slice 1), the middle slice (slice 168), and the most proximal slice (slice 336). The scale bars are 5 mm.

| **Table S1.** *Spasticity in the CP cohort*. Spasticity in the lower limbs was described using the Modified Ashworth Scale. The table shows the mode and range of values for the Modified Ashworth Scale for each muscle group. Additionally, the distribution of values (number of participants with the score) from 0 to 4 on the scale is shown for each muscle group. Abbreviations: L, left; R, right. |
| --- |
| 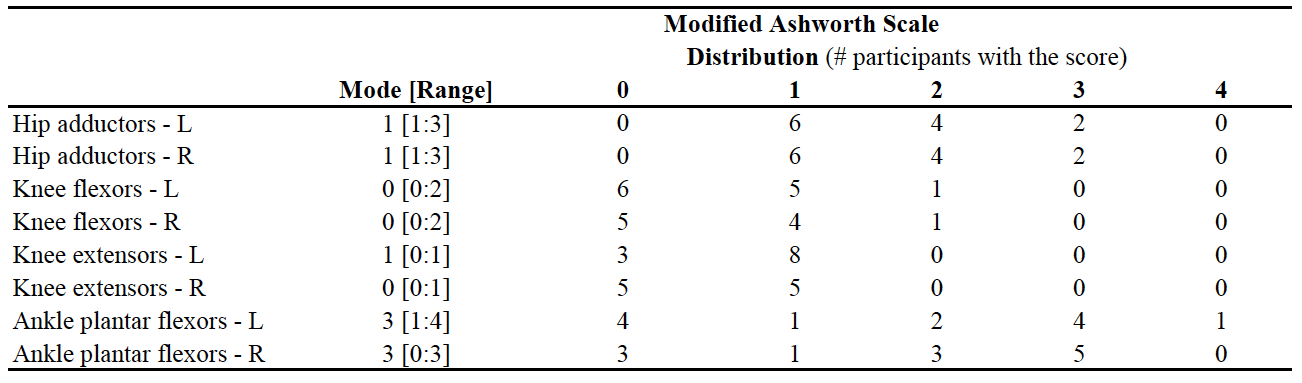 |

| **Table S2.** **Correlation coefficients between bone density and muscle spasticity.** Associations between DXA measures of aBMD and Z-scores at the proximal and lateral distal femur with measures of lower limb spasticity using the Modified Ashworth Scale. The Modified Ashworth Scale describes the degree of spasticity in the muscle group on an ordinal scale from 0 to 4. Data are shown as the correlation coefficient (p-value). Kendall’s tau was performed to explore associations between continuous bone parameters and ordinal Modified Ashworth Scale. |
| --- |
| *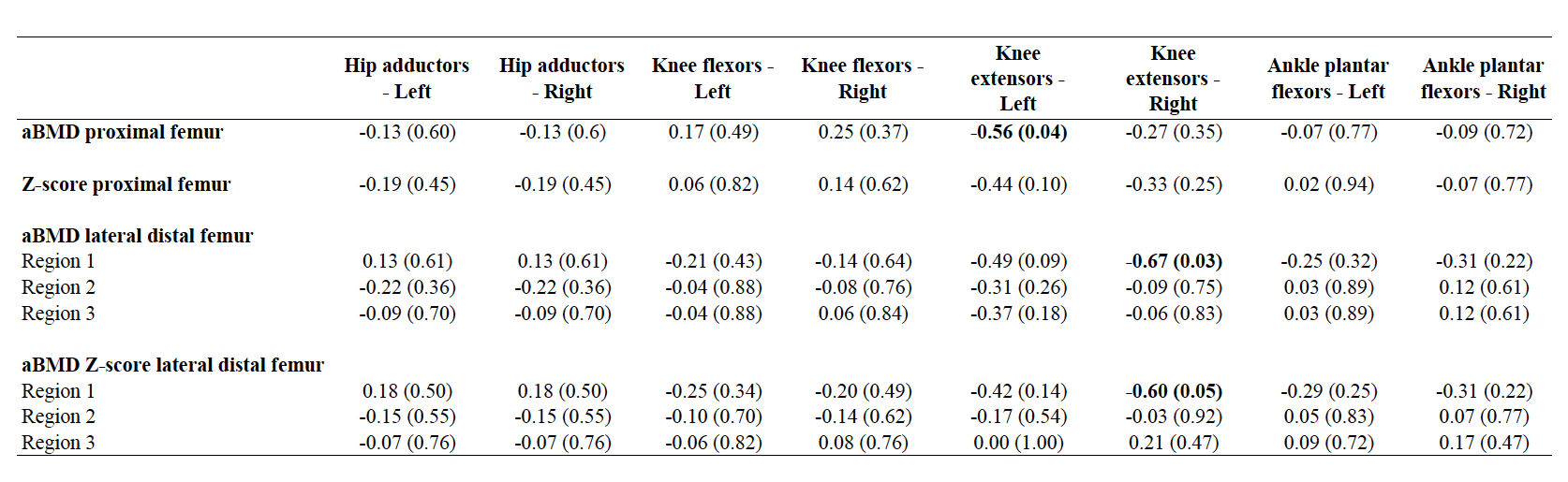* |

**Table S3:** **HR-pQCT data at metaphyseal radius.** Data for the distal and proximal radius stacks are presented as unadjusted mean $\pm$standard deviation. An independent t-test or a Mann-Whitney test was performed. ANCOVA was performed to adjust the HR-pQCT parameters for participant height. (if ANCOVA assumptions were violated, the adjusted p-values are not reported). Abbreviation: NR: not reported.

|  | | | | **Control group** | | | | | **CP group** | | | | | | | | **P value** | | **P value adjusted for height** | |  |  |  |
| --- | --- | --- | --- | --- | --- | --- | --- | --- | --- | --- | --- | --- | --- | --- | --- | --- | --- | --- | --- | --- | --- | --- | --- |
|  |  |  |  |  |  |  |  |  |  |  |  |  |  |  |  |  |  |  |  |  |  | | |
|  |  |  |  | | |  |  | | |  | |  | |  | |  | |  | |  | |  |  |
| **Metaphyseal radius – distal stack** | |  | n = 7 | | | | | | | n = 4 | | | | | |  | |  | |  | |  |  |
| Total area | Tt.Ar | mm^2^ | 108.3 | | | ± | 16.7 | | | 104.7 | | ± | | 24.0 | | 0.77 | | 0.15 | |  | |  |  |
| Total vBMD | Tt.vBMD | mg HA/cm^3^ | 294.6 | | | ± | 31.4 | | | 274.4 | | ± | | 58.3 | | 0.47 | | 0.17 | |  | |  |  |
| Cortical vBMD | Ct.vBMD | mg HA/cm^3^ | 736.2 | | | ± | 27.5 | | | 655.4 | | ± | | 78.5 | | **0.03** | | NR | |  | |  |  |
| Cortical area | Ct.Ar | mm^2^ | 26.9 | | | ± | 4.3 | | | 25.4 | | ± | | 0.7 | | 0.52 | | 0.92 | |  | |  |  |
| Cortical thickness | Ct.Th | mm | 0.75 | | | ± | 0.09 | | | 0.74 | | ± | | 0.10 | | 0.89 | | 0.43 | |  | |  |  |
| Cortical porosity | Ct.Po | mm^3^/mm^3^ | 0.004 | | | ± | 0.002 | | | 0.003 | | ± | | 0.001 | | 0.32 | | NR | |  | |  |  |
| Trabecular vBMD | Tb.vBMD | mg HA/cm^3^ | 156.3 | | | ± | 27.0 | | | 146.2 | | ± | | 26.0 | | 0.56 | | 0.82 | |  | |  |  |
| Trabecular area | Tb.Ar | mm^2^ | 83.6 | | | ± | 13.8 | | | 81.5 | | ± | | 24.2 | | 0.86 | | 0.11 | |  | |  |  |
| Trabecular bone volume fraction | Tb.BV/TV | mm^3^/mm^3^ | 0.192 | | | ± | 0.044 | | | 0.168 | | ± | | 0.038 | | 0.39 | | 0.72 | |  | |  |  |
| Trabecular number | Tb.N | 1/mm | 1.580 | | | ± | 0.197 | | | 1.416 | | ± | | 0.214 | | 0.23 | | 0.65 | |  | |  |  |
| Trabecular thickness | Tb.Th | mm | 0.201 | | | ± | 0.009 | | | 0.207 | | ± | | 0.007 | | 0.27 | | 0.26 | |  | |  |  |
| Trabecular separation | Tb.Sp | mm | 0.611 | | | ± | 0.088 | | | 0.702 | | ± | | 0.117 | | 0.18 | | 0.52 | |  | |  |  |
|  |  |  |  | | |  |  | | |  | |  | |  | |  | |  | |  | |  |  |
|  | | | | | | | | | | | | | | | | | | |  | |  | | |
| **Metaphyseal radius – proximal stack** | | | | | n | = | | 7 | | | n | | = | | 4 | |  | |  | |  | |  |
| Total area | Tt.Ar | mm^2^ | 59.6 | | | ± | 18.7 | | | 62.2 | | ± | | 12.0 | | 0.82 | | 0.06 | |  | |  |  |
| Total vBMD | Tt.vBMD | mg HA/cm^3^ | 462.1 | | | ± | 50.9 | | | 417.8 | | ± | | 71.7 | | 0.26 | | 0.13 | |  | |  |  |
| Cortical vBMD | Ct.vBMD | mg HA/cm^3^ | 845.6 | | | ± | 30.4 | | | 798.1 | | ± | | 35.5 | | **0.04** | | 0.08 | |  | |  |  |
| Cortical area | Ct.Ar | mm^2^ | 33.2 | | | ± | 4.8 | | | 29.2 | | ± | | 1.6 | | 0.14 | | NR | |  | |  |  |
| Cortical thickness | Ct.Th | mm | 1.27 | | | ± | 0.14 | | | 1.18 | | ± | | 0.10 | | 0.29 | | 0.21 | |  | |  |  |
| Cortical porosity | Ct.Po | mm^3^/mm^3^ | 0.011 | | | ± | 0.003 | | | 0.008 | | ± | | 0.006 | | 0.40 | | NR | |  | |  |  |
| Trabecular vBMD | Tb.vBMD | mg HA/cm^3^ | 97.0 | | | ± | 36.8 | | | 82.3 | | ± | | 32.3 | | 0.53 | | 0.59 | |  | |  |  |
| Trabecular area | Tb.Ar | mm^2^ | 35.0 | | | ± | 6.7 | | | 34.7 | | ± | | 10.9 | | 0.96 | | 0.08 | |  | |  |  |
| Trabecular bone volume fraction | Tb.BV/TV | mm^3^/mm^3^ | 0.124 | | | ± | 0.045 | | | 0.103 | | ± | | 0.030 | | 0.42 | | 0.64 | |  | |  |  |
| Trabecular number | Tb.N | 1/mm | 1.035 | | | ± | 0.352 | | | 0.755 | | ± | | 0.277 | | 0.21 | | 0.94 | |  | |  |  |
| Trabecular thickness | Tb.Th | mm | 0.175 | | | ± | 0.017 | | | 0.180 | | ± | | 0.019 | | 0.68 | | 0.07 | |  | |  |  |
| Trabecular separation | Tb.Sp | mm | 1.051 | | | ± | 0.483 | | | 1.425 | | ± | | 0.422 | | 0.23 | | 0.97 | |  | |  |  |

**Table S4:** **HR-pQCT data at metaphyseal tibia.** Data for the distal and proximal tibial stacks are presented as unadjusted mean $\pm$standard deviation. An independent t-test or a Mann-Whitney test was performed. ANCOVA was performed to adjust the HR-pQCT parameters for participant height. (if ANCOVA assumptions were violated, the adjusted p-values are not reported). Abbreviation: NR: not reported.

|  | | | | **Control group** | | | | | **CP group** | | | | | | | | **P value** | | **P value adjusted for height** | |  |  |
| --- | --- | --- | --- | --- | --- | --- | --- | --- | --- | --- | --- | --- | --- | --- | --- | --- | --- | --- | --- | --- | --- | --- |
|  |  |  |  |  |  |  |  |  |  |  |  |  |  |  |  |  |  |  |  |  |  | |
|  |  |  |  | | |  |  | | |  | |  | |  | |  | |  | |  |  |  |
| **Metaphyseal tibia – distal stack** |  |  | n = 7 | | | | | | | n = 4 | | | | | |  | |  | |  |  |  |
| Total area | Tt.Ar | mm^2^ | 316.7 | | | ± | 49.2 | | | 202.7 | | ± | | 28.1 | | **0.002** | | **0.05** | |  |  |  |
| Total vBMD | Tt.vBMD | mg HA/cm^3^ | 235.0 | | | ± | 41.1 | | | 212.4 | | ± | | 23.8 | | 0.53 | | NR | |  |  |  |
| Cortical vBMD | Ct.vBMD | mg HA/cm^3^ | 740.2 | | | ± | 49.3 | | | 697.0 | | ± | | 40.2 | | 0.17 | | 0.51 | |  |  |  |
| Cortical area | Ct.Ar | mm^2^ | 49.2 | | | ± | 10.0 | | | 38.7 | | ± | | 3.5 | | 0.08 | | NR | |  |  |  |
| Cortical thickness | Ct.Th | mm | 0.80 | | | ± | .16 | | | 0.82 | | ± | | 0.03 | | 0.23 | | 0.48 | |  |  |  |
| Cortical porosity | Ct.Po | mm^3^/mm^3^ | 0.007 | | | ± | 0.004 | | | 0.006 | | ± | | 0.003 | | 0.71 | | NR | |  |  |  |
| Trabecular vBMD | Tb.vBMD | mg HA/cm^3^ | 145.4 | | | ± | 21.8 | | | 103.1 | | ± | | 27.9 | | **0.01** | | 0.08 | |  |  |  |
| Trabecular area | Tb.Ar | mm^2^ | 271.2 | | | ± | 45.5 | | | 166.9 | | ± | | 25.0 | | **0.002** | | 0.06 | |  |  |  |
| Trabecular bone volume fraction | Tb.BV/TV | mm^3^/mm^3^ | 0.195 | | | ± | 0.036 | | | 0.134 | | ± | | 0.039 | | **0.02** | | 0.14 | |  |  |  |
| Trabecular number | Tb.N | 1/mm | 1.445 | | | ± | 0.130 | | | 0.993 | | ± | | 0.252 | | **0.03** | | **0.02** | |  |  |  |
| Trabecular thickness | Tb.Th | mm | 0.223 | | | ± | 0.010 | | | 0.220 | | ± | | 0.009 | | 0.65 | | 0.99 | |  |  |  |
| Trabecular separation | Tb.Sp | mm | 0.668 | | | ± | 0.068 | | | 1.071 | | ± | | 0.312 | | 0.08 | | NR | |  |  |  |
|  |  |  |  | | |  |  | | |  | |  | |  | |  | |  | |  |  |  |
|  | | | | | | | | | | | | | | | | | | |  | |  | |
| **Metaphyseal tibia – proximal stack** | | | | | n | = | | 7 | | | n | | = | | 5 | |  | |  | |  |  |
| Total area | Tt.Ar | mm^2^ | 212.4 | | | ± | 67.5 | | | 139.6 | | ± | | 17.5 | | **0.05** | | 0.60 | |  |  |  |
| Total vBMD | Tt.vBMD | mg HA/cm^3^ | 307.6 | | | ± | 47.2 | | | 307.5 | | ± | | 24.5 | | 0.53 | | 0.83 | |  |  |  |
| Cortical vBMD | Ct.vBMD | mg HA/cm^3^ | 839.8 | | | ± | 45.2 | | | 800.9 | | ± | | 21.1 | | 0.11 | | 0.40 | |  |  |  |
| Cortical area | Ct.Ar | mm^2^ | 64.2 | | | ± | 10.7 | | | 49.7 | | ± | | 4.9 | | **0.02** | | NR | |  |  |  |
| Cortical thickness | Ct.Th | mm | 1.29 | | | ± | 0.17 | | | 1.31 | | ± | | 0.07 | | 0.15 | | 0.32 | |  |  |  |
| Cortical porosity | Ct.Po | mm^3^/mm^3^ | 0.015 | | | ± | 0.014 | | | 0.015 | | ± | | 0.012 | | 0.76 | | NR | |  |  |  |
| Trabecular vBMD | Tb.vBMD | mg HA/cm^3^ | 104.5 | | | ± | 24.9 | | | 44.2 | | ± | | 16.5 | | **0.003** | | **0.02** | |  |  |  |
| Trabecular area | Tb.Ar | mm^2^ | 165.7 | | | ± | 26.8 | | | 92.2 | | ± | | 13.4 | | **<.001** | | **0.01** | |  |  |  |
| Trabecular bone volume fraction | Tb.BV/TV | mm^3^/mm^3^ | 0.147 | | | ± | 0.037 | | | 0.084 | | ± | | 0.018 | | **0.003** | | 0.12 | |  |  |  |
| Trabecular number | Tb.N | 1/mm | 1.098 | | | ± | 0.190 | | | 0.464 | | ± | | 0.133 | | **<.001** | | **0.003** | |  |  |  |
| Trabecular thickness | Tb.Th | mm | 0.217 | | | ± | 0.015 | | | 0.202 | | ± | | 0.019 | | 0.15 | | 0.57 | |  |  |  |
| Trabecular separation | Tb.Sp | mm | 0.914 | | | ± | 0.153 | | | 2.372 | | ± | | 0.704 | | **0.01** | | NR | |  |  |  |
